# Inhibitory effect of alpinetin on IL‐6 expression by promoting cytosine methylation in CpG islands in the IL‐6 promoter region

**DOI:** 10.1002/mgg3.993

**Published:** 2019-11-13

**Authors:** Ke Hu, Yuxian Li, Minghua Liang, Lijing Liu, Yuefu Chen, Minjiang Huang, Bifeng Tan, Yingquan Luo, Huiming Yin

**Affiliations:** ^1^ Medical college Hunan University of Medicine Huaihua Hunan PR China; ^2^ Department of Pediatrics first people's hospital of huaihua Huaihua Hunan PR China; ^3^ Department of Cardiology first affiliated hospital Hunan University of Medicine Huaihua Hunan PR China; ^4^ Department of Senile Disease second Xiangya hospital Central South University Changsha Hunan PR China; ^5^ Department of Respiration first affiliated hospital Hunan University of Medicine Huaihua Hunan PR China

**Keywords:** Alpinetin, CpG island, IL‐6, methylation, murine macrophage, promoter region

## Abstract

**Background:**

Alpinetin is a flavonoid which exerts antibacterial and anti‐inflammatory functions. In order to prove that the induced methylation is an important mechanism for alpinetin in regulating the expression of inflammatory factor Interleukin‐6 (IL‐6), we detected the dinucleotide methylation status of CpG islands in the IL‐6 promoter region and IL‐6 level after treatment of RAW246.7 murine macrophages with alpinetin.

**Methods:**

After RAW246.7 murine macrophages were treated with alpinetin, alpinetin + GW9662 (the peroxisome proliferator‐activated receptor (PPAR) antagonist), and alpinetin + DNA methyltransferase 3 alpha (DNMT3A) siRNA for 96 hr, CpG islands were analyzed using time‐of‐flight mass spectrophotometry (TOF‐MS) and bisulfite sequencing polymerase chain reaction (BSP). Dinucleotide methylation status of the CpG islands in the IL‐6 promoter region was analyzed by methylation‐specific Polymerase Chain Reaction (PCR). IL‐6 level was detected using the enzyme‐linked immunosorbent assay (ELISA) method. Pearson's correlation analysis was conducted to test for potential correlation between the methylation status of CpG islands in the IL‐6 promoter region and IL‐6 level in RAW 246.7 cells.

**Results:**

Alpinetin promoted dinucleotide methylation status of two CpG islands in the IL‐6 promoter region stretching 500–2500 bp upstream of the transcriptional start site (TSS) (*p* < .05). This promoting effect was more significant for the CpG island stretching 500–1500 bp long. The methylation ratio of dinucleotide at this position was significantly inversely correlated with the level of IL‐6 (*p* < .05). PPAR antagonist GW9662 and interference of DNMT3A could reverse both the alpinetin‐induced methylation and inhibitory effects on IL‐6 expression.

**Conclusion:**

Alpinetin could induce dinucleotide methylation status of CpG islands in the IL‐6 promoter region by activating methyltransferase, thus inhibiting IL‐6 expression in murine macrophages.

## Graphical abstract

We found Dinucleotide methylation status of CpG islands in the IL‐6 promotor region correlated ith IL‐6 expression in murine macrophages. Alpinetin could induce dinucleotide methylation status of CpG islands in the IL‐6 promotor region by activating methyltransferase, thus inhibiting IL‐6 expression.

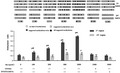

## 1 INTRODUCTION

Inflammatory damage is considered as the main threat to human health, which is intended to restore the steady‐state level of inflammatory factors (Abdelhalim, Moussa, Qaid, & Al‐Ayed, 2018). Glucocorticoids were once used as an intervention for inflammatory diseases including rheumatoid arthritis and inflammatory bowel disease, but its side effects of long‐term use cannot be ignored (Lambert, Roff, Panganiban, Douglas, & Ishmael, 2018; Palme, 2018). Therefore, looking for new anti‐inflammatory drugs with low toxicity is the primary concern at present.

Chinese patent medicines have proven to have anti‐inflammatory functions, and many new herbal medicines have drawing an increasing attention because of its anti‐inflammatory effects (Hu, Yang, Tu, Luo, & Ma, 2013; Lee & Lee, 2016; Liang et al., 2018; Raja, Saranya, & Prabhu, 2019; Tsai et al., 2018; Yang et al., 2019). For example, a Dong medicine extracted from the fruits of rusty‐leaf muuna is usually used to treat painful swelling on the body surface. Evidences further prove that 2‐phenyl‐chromone, a type of flavonoid, is the main active component in this medicine (Tsai et al., 2018). Flavonoids have drawn an increasing attention due to their roles in regulating glucose and lipid metabolism, and insulin resistance (Raja et al., 2019; Yang et al., 2019). Flavonoids may also play a part in regulating the production of inflammatory mediators (Lee & Lee, 2016). The pharmacological effect of flavonoids is related to the activation of PPARs which inhibits the expression of inflammatory mediators through several pathways. Among the known flavonoids, alpinetin derived from Alpinia katsumadai Hayata is the most easily available and highly effective in activating PPAR (Hu et al., 2013). Previous study indicated that alpinetin inhibits the expressions of intracellular inflammatory signaling pathways after activating PPARs, while inhibit the synthesis of upstream transcriptional factors of inflammatory genes such as tumor necrosis factor α (TNF‐α), IL‐1ß, and IL‐6. Notably, alpinetin induces deacetylation of H3K9 that binds to the promoter region of the inflammatory genes by activating histone deacetylase 1 (HDAC1), which further influences the binding of the transcriptional factors to the promoter (Liang et al., 2018). Additionally, alpinetin regulates the expression of the inflammatory mediators TNF‐α, IL‐1β as well as Toll‐like receptor 4 (TLR4) mediated nuclear transcription factor‐kappaB (NF‐κB) and NOD‐like receptor protein 3 (NLRP3) inflammasome activation, indicating that alpinetin has protective effects on DSS dextran sulfate sodium (DSS)‐induced colitis in mice (He et al., 2016). In our previous report, it was found that the level of DNMT3A binding to PPAR intranuclearly detected by the co‐immunoprecipitation technology is promoted with the increase of the concentration of alpinetin in RAW246.7 murine macrophages. This study indicated that alpinetin may regulate the expressions of the target genes by inducing methylation after activating PPAR (Liang et al., 2016).

In the present study, we detected the effects and mechanisms of alpinetin on dinucleotide methylation status of CpG islands in the IL‐6 promoter region and IL‐6 level in RAW246.7 murine macrophages, for providing the basis for the clinical use of alpinetin.

## 2 MATERIALS AND METHODS

### 2.1 Cell culture

RAW246.7 murine macrophages were purchased from Nanjing Hua'ao Biotechnology Co., Ltd., China, and cultured in the RPIM1640 medium (Promega Biosciences Inc.) containing 10% fetal bovine serum (FBS), 100 U/ml penicillin and 0.1 mg/ml streptomycin (Invitrogen, Carlsbad, CA, USA) at 37°C in a 5% CO_2_ incubator. The culture medium was replaced on a regular basis and cell passage was performed.

### 2.2 Treatment

RAW246.7 murine macrophages of log phase were inoculated to the plates at a dose of 1 × 10^6^ cells per well. The following groups included: alpinetin group (0, 50, 100, 200, 500, 1,000 μg/ml), alpinetin (1,000 μg/ml) + GW9662 (PPAR antagonist, 0.1 mmol/ml) group, and alpinetin (1,000 μg/ml) + DNMT3A siRNA (1 mmol/L) group. An equal volume of RPIM1640 was added into the control group. In alpinetin group, different concentrations of alpinetin (0, 50, 100, 500, and 1,000 μg/ml) were used to treat with cells for 24 hr. In alpinetin (1,000 μg/ml) + GW9662 group, cells were treated with 0.1 mmol/ml GW9662 for 24 hr, followed by 1,000 μg/ml alpinetin stimulation for 24 hr. In alpinetin + DNMT3A siRNA group, cells were first transfected with 1 mmol/L DNMT3AsiRNA for 24 hr and then treated with 1,000 μg/ml alpinetin for 24 hr. Alpinetin was purchased from Nanjing Zelang Biotechnology Co., Ltd., China. GW9662 was provided by Sangon Biotech (Shanghai, China) Co., Ltd., China.

### 2.3 Transfection

After cells reached 65% confluency, DNMT3A siRNA transfection was done the manufacturer's instructions. Before the transfection, 2 μL Lipofectamine 2000 (Promega Biosciences Inc.) was mixed with 50 μL Opti‐MEM (Promega Biosciences Inc.) and left to stand for 5 min. Then the cells were mixed with Opti‐MEM‐diluted DNMT3A siRNA (1 mmol/L). After standing for 20 min, the mixture was added into the wells containingRAW246.7 cells and cultured at 37°C in a 5% CO_2_ incubator for 24 hr, then cells and culture medium were collected. DNMT3A siRNA (5’‐CGGACCACCTTACGTGACC‐3’) was constructed by Wuhan Genesil Biotechnology Co., Ltd., China.

### 2.4 DNA Modification

Wizard genomic DNA purification kits (Promega Biosciences Inc.) were used to extract DNA from RAW246.7 murine macrophages. DNA Methylation Gold kits (Promega Biosciences Inc.) were used for sodium bisulfate modification of DNA according to the instruction manual. The unmethylated cytosine was converted into uracil after sodium bisulfate modification, while the methylated cytosine remained unchanged.

### 2.5 Bisulfite sequencing polymerase chain reaction (BSP) detection

The sequence of murine IL‐6 gene was searched at the University of California Santa Cruz (UCSC) (://genome.ucsc.edu/cgi-bin/hggateway). The sequence stretching 3,000 bp upstream of the transcriptional start site (TSS) was located at the Genomic Sequence interface and input into the on‐line website Cpgplot. It was confirmed that the two CpG islands including 500–1500 bp and 1500–2500 bp upstream of the TSS in the IL‐6 promoter region which met the definition of CpG islands: longer than 200 bp, GC content approaching 50%, and expected ObsCpG/ExpCpG exceeding 0.50.

Primers were designed based on the sequences flanking the two CpG islands in the IL‐6 promoter region using the Methyl Primer Express V2.0 software (Applied Biosystems, Foster City, CA, USA). Two pairs of upstream and downstream primers were designed (Table 1). The amplified region was located in each CpG island and the upstream and downstream primers contained CpG dinucleotide. According to the instruction manual of TaKaRa, Japan, the PCR reaction system was 50 μL in volume containing 1 μl of template for sodium bisulfate modification of DNA, 1 μL upstream and downstream primer each, 5 μl of 10 × PCR buffer containing Mg^2+^, 1 μl of 10 mol/L dNTP, and 0.8 μl of 5 × 10^6^ U/L Taq DNA polymerase. PCR procedures was: predenaturation at 95°C for 4 min, denaturation at 94°C for 30 s, annealing at 55°C for 30 s, extension at 72°C for 30 s, 38 cycles, and final extension at 72°C for 8 min. PCR products were analyzed by 3% agarose gel electrophoresis. Target fragments were identified and 10 μl of the products was submitted to China National GeneBank (Shenzhen, China) for TOF‐MS. According to the principle of modification, if the cytosine in the original sequence is methylated, the sequencing result remains unchanged; if the cytosine in the original sequence is not methylated, the sequencing will indicate that cytosine is converted into thymine (T). Each batch of DNA was treated in three replicates. The above CpG sites were screened and lined up in order. Different colors were used to indicate the sequencing results. The methylation ratio was calculated for the CpG sites in the amplified region.

**Table 1:** Sequence of primers used in amplification of CpG islands in IL‐6 promoter region in BSP test

Number of island	Position (distance from TSS)	Primer sequence (5’−3’)	Region size
1st	500−1500bp	F:5’‐ACTCTAATCGCCTGTGTGTTT−3’ R:5’‐TCTGATCTGAAGCAACTTAGG−3’	1038bp
2nd	1500−2500bp	F:5’‐GCATCAGAACCCCAGGAACA−3’ R:5’‐CACAAGAATCAACCAGCTTTT−3’	991bp

### 2.6 Methylation specific polymerase chain reaction (MSP) detection

Cpgplot library was searched. One pair of methylated and unmethylated primers were designed for MSP using Methyl Primer Express V 1.0 (Applied Biosystems Inc.) according to the sequences at 500–1500 bp and 1500–2500 bp upstream of the TSS in the IL‐6 promoter region. Primers were synthesized by Sangon Biotech (Shanghai) Co., Ltd., China, and shown in Table 2. PCR was performed using methylated and unmethylated primers, respectively. The PCR reaction system was 20 μl in volume, containing 1 μl of template for sodium bisulfate modification of DNA, 1 μl upstream and downstream primer each, 5 μl of 10 × PCR buffer containing Mg^2+^, 1 μl of 10 mol/L dNTP, and 0.8 μl of 5 × 10^6^ U/L Taq DNA polymerase. The volume was diluted to 20 μl using double distilled water. PCR reaction procedures were as follows: predenaturation at 95°C for 5 min, denaturation at 95°C for 100 s, annealing at 56°C for 10 s, extension at 72°C for 30 s, final extension at 72°C for 30 s, 36 cycles, and final extension at 72°C for 5 min. Then 10 μl of the PCR product was analyzed by 3% agarose gel electrophoresis. The gels were scanned using an imaging system. Target bands in the gel represented the methylation status of CpG islands in the primers. Optical densities of target bands were calculated using AlphaEase FC Version 4 software (AlphalmagerHP, Alpha Innotech) to reflect the relative methylation ratio.

**Table 2:** Sequence of primers used in amplification of CpG islands locating at IL‐6 promoter in MSP test

Number of island	Primer sequence (5’−3’)	Region size
1st	**Methylated**: F:5’‐TCTGGCGGCAGTGGGATCGGCAC−3’ R:5’‐GCTATACAGGTCCGGTGCTGGA−3’ **Non‐methylated**: F:5’‐TCTGGTGGCAGTGGGATTGGCAC−3’ R:5’‐GCTATACAGGTCTGGTGCTGGA−3’	325bp
2nd	**Methylated**: F:5’‐AATCGTTAAGCAGCGAAAAGAAA−3’ R:5’‐CTAGTTTATTCGCCTTCTGATTT−3’ **Nonmethylated**: F:5’‐AATTGTTAAGCAGTGAAAAGAAA−3’ R:5’‐CTAGTTTATTTGCCTTCTGATTT−3’	336bp

### 2.7 Western blot analysis

A total of 1 × 10^6^ RAW246.7 cells with high viability were added with RLN lysis buffer containing 0.1 mol/L Tris‐HCl, 150 mmol/L NaCl, 1.5 mmol/L MgCl_2_ and 0.5% Nonidet to obtain nuclear precipitate. Radio immunoprecipitation assay (RIPA) lysis buffer (Sangon Biotech (Shanghai) Co., Ltd., China) containing 50 mmol/L Tris‐HCl, 150 mmol/L NaCl, 1% Triton X‐100 and 1% sodium deoxycholate was added and oscillated. Centrifugation was performed and the supernatant was collected to obtain nuclear protein extract. After sodium dodecyl sulfate‐polyacrylamide gel electrophoresis (SDS‐PAGE), 30 g proteins were transferred to membranes and sealed with skimmed milk powder in Tris Buffered saline Tween (TBST) for 24 hr. Next, the membranes were incubated with labeled primary antibodies against PPAR and DNMT3A (all dilution at 1:1,000, Cell Signaling Technology, Danvers, MA, USA) at room temperature for 2 hr. The membranes were washed with TBST for three times, further incubated with HRP‐labeled goat anti‐mouse secondary antibody (1:1,000, Cell Signaling Technology) and washed with TBST for three times. Enhanced chemiluminescence (ECL) substrate was added for color development. Optical density (OD) of the target band was calculated using AlphaEase FC Version 4 software (AlphalmagerHP, Alpha Innotech), and the result was expressed as the grayscale ratio between the target protein and internal reference (β‐actin).

### 2.8 Determination of DNA methyltransferase 3 alpha (DNMT3A) activity

DNMT3A catalyzed tyrosine receiving a methyl group donated by s‐adenosylmethionine (SAM) to form 3‐methyltyrosine. We obtained 25 μL of nuclear protein extract using 50 mmol/L 4‐(2‐Hydroxyethyl)‐1‐piperazineethanesulfonic acid (HEPES), 0.2 mmol/L MnCl_2_, 2mmol/L SAM and 2 mmol/L DTT following the steps in WB analysis. Thus, the methylation reaction system of 200 μl was established. The reaction time was 10–30 min, and 50 μl of the reaction liquid was taken out every 5 min. The reaction was terminated by adding trifluoroacetic acid (TFA) and the reaction liquid was subjected to high performance liquid chromatography (HPLC). The first peak appearing in HPLC corresponded to tyrosine; the second peak occurring at 2.5–10 min after the first peak corresponded to 3‐methyltyrosine. The peak height ratio between 3‐ methyltyrosine and tyrosine was the relative activity of DNMT3A.

### 2.9 Enzyme‐linked immunosorbent assay (ELISA) measurement

IL‐6 level in the culture medium was determined by ELISA kits (Beijing Huanya Taike Company, China) according the instruction. Standard wells, sample wells and blank control wells were set up. A total of 10 μl of the standard and sample was added into each well, respectively, and cultured at 37°C for 30 min. The coated ELISA plate was washed and ELISA working solution was added. The plates were washed three times and incubated with ECL substrate at 37°C in the dark for 15 min for color development. The reaction was terminated by adding the stopping solution. IL‐6 level was measured three times per well by plotting the standard curve, and the average was taken (pg/ml).

### 2.10 Statistical analysis

Measurements were expressed as mean ± standard deviation (*SD*). All statistical analyses were conducted using Statistical Package for the Social Sciences (SPSS) 19.0 software (IBM Corp, Armonk, NY, USA). Multiple comparisons were performed using one‐way analysis of variance (ANOVA) Pairwise comparisons were conducted using least significant difference (LSD) test. The strength of correlation between the dinucleotide methylation status of the CpG islands in the IL‐6 promoter region and IL‐6 level was measured by Pearson's correlation coefficient. The difference was considered significant when *p* < .05.

## 3 RESULTS

### 3.1 CpG Islands located at the IL‐6 promoter region in mice retrieved from bioinformatics databases

**Figure 1:**
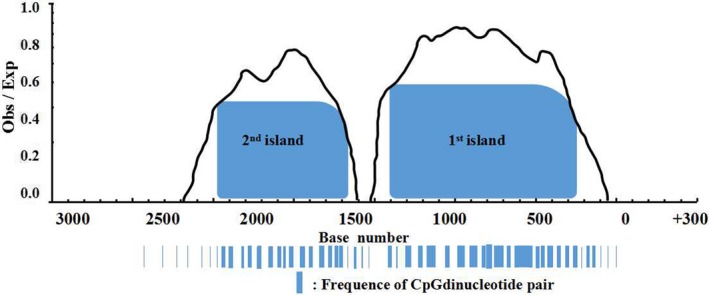
CpG Islands located at IL‐6 promoter site retrieved from UCSC and Cpgplot

As shown in Figure 1, two stretches of 500–1500 and 1500–2500 bp upstream of TSS were considered on CpG islands, respectively. Location of the islands and density of CpG dinucleotides described that the 500–1500 bp region was defined as the first CpG island containing 70 CpG dinucleotide pairs, while 1500–2500 bp was the second CpG island containing 46 CpG dinucleotide pairs.

### 3.2 Alpinetin promoted methylation on CpG islands in IL‐6 promoter region via activation of PPAR and DNMT3A

**Figure 2:**
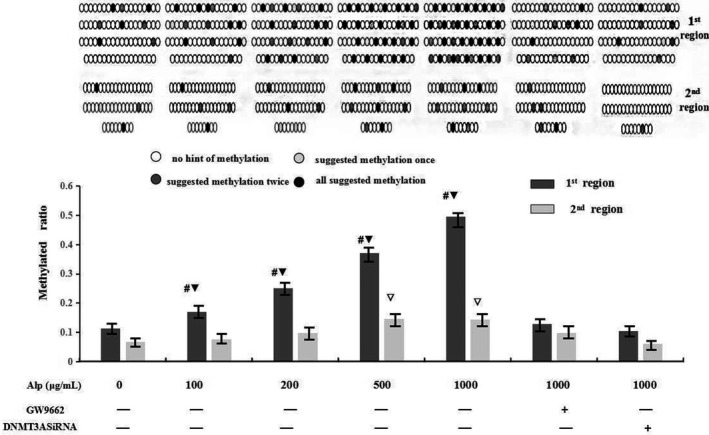
Methylation status of CpG dinucleotide at CpG island in IL‐6 promotor region tested by BSP combined with TOF‐MS. In this test, CpG dinucleotide pairs stretching 500–2500 bp upstream of TSS of IL‐6 were all selected and lined up in order. Each site was sequenced for three times, and different colors were used to describe the result: If the site was confirmed as cytosine for three times, this locus would be represented by black; light or dark grey respectively represented confirmation for twice or once. Finally, white was used if thymine was indicated for three times. ^#,▽^ *p* < .05 versus control group (#,▽respectively denoted the first and second CpG island); ^▼^ *p* < .05 versus the second CpG island

Results in Figure 2 suggested that, in the control group, the methylation ratio of CpG at the two CpG islands remained at a low level (11.4% in the first island and 6.8% in the second island). However, alpinetin displayed a promotion in the methylation ratio of the two CpG islands, especially in the first CpG island (500–1500 bp upstream of TSS) in a dose‐dependent manner. Furthermore, the promoting effect could be reversed by PPAR blocker GW9662 or DNMT3A interference.

**Figure 3:**
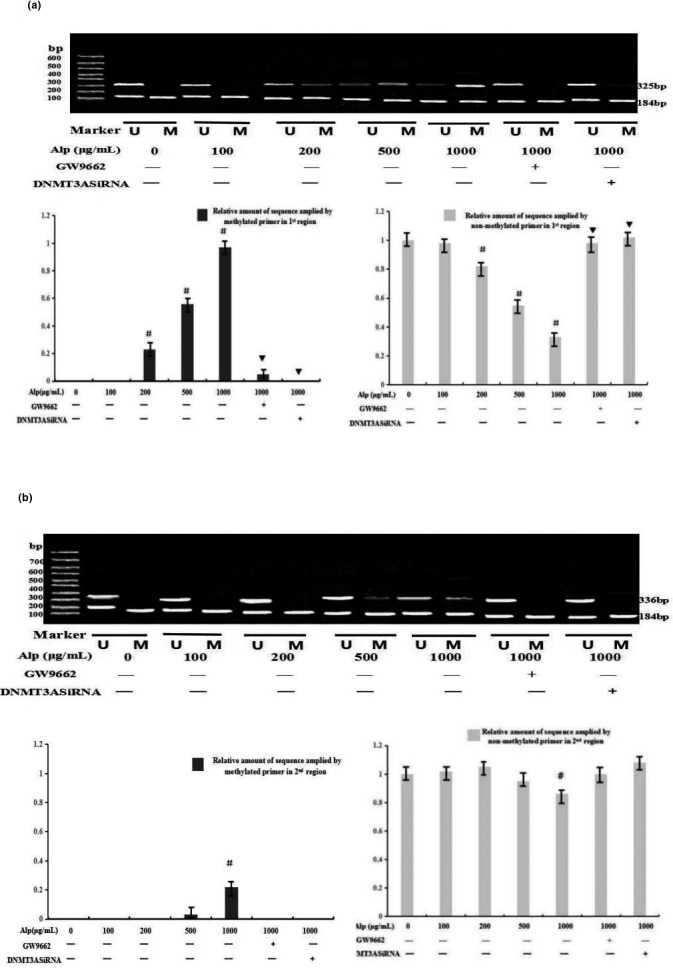
Methylation ratio of CpG dinucleotide at two CpG islands in the IL‐6 promotor region evaluated by MSP. Following treatment with alpinetin at 0, 50, 100, 200, 500, 1,000 g/ml, the methylation ratios of CpG dinucleotide at the first (a) and the second (b) CpG islands in the IL‐6 promotor region were evaluated by MSP. ^#^ *p* < .05 versus control group

In addition, the methylation ratio of CpG dinucleotide in the first and second CpG islands was evaluated by MSP (Figure 3). It was shown that alpinetin increased the relative amount of sequence (located at the first island) when amplified by methylated primers in a dose‐dependent manner, while a completely opposite result was obtained when amplified by nonmethylated primers (*p* < .05, Figure 3a). Moreover, the dose‐effect relationship between alpinetin and methylation was not so obvious at the second CpG island (*p* > .05). Only the content at a higher concentration (1 mg/ml) amplified by primers could be affected by alpinetin. Furthermore, changes in methylation status could also be reversed by the use of PPAR blocker GW9662 or DNMT3A interference (*p* < .05, Figure 3b).

Therefore, above findings suggested that the increase in methyltransferase activity induced by alpinetin may be attributed to the PPAR and DNMT3A activation.

### 3.3 Alpinetin inhibited IL‐6 production via PPAR/DNMT3A pathway

**Figure 4:**
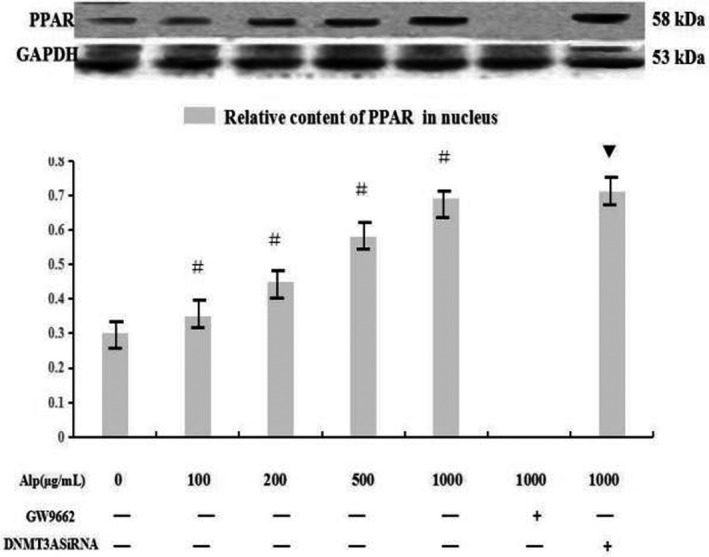
Effect of alpinetin on protein expression of PPAR. Expressions of PPAR were determined by Western blot assay after cells were treated with alpinetin at different concentrations. Data were expressed as means ± *SD*. ^#^ *p* < .05 versus control group, ^▼^ *p* < .05 versus 1 mg/ml Alp group

Following treatment of alpinetin, protein expression of PPAR in the RAW246.7 cells was tested by Western blot assay. Results showed that alpinetin up‐regulated the expression of PPAR in a dose‐dependent manner but this increase could be blocked by the use of PPAR inhibitor GW9662 (*p* < .05). Interference of DNMT3A had no influence on PPAR expression (*p* > .05) (Figure 4).

**Figure 5:**
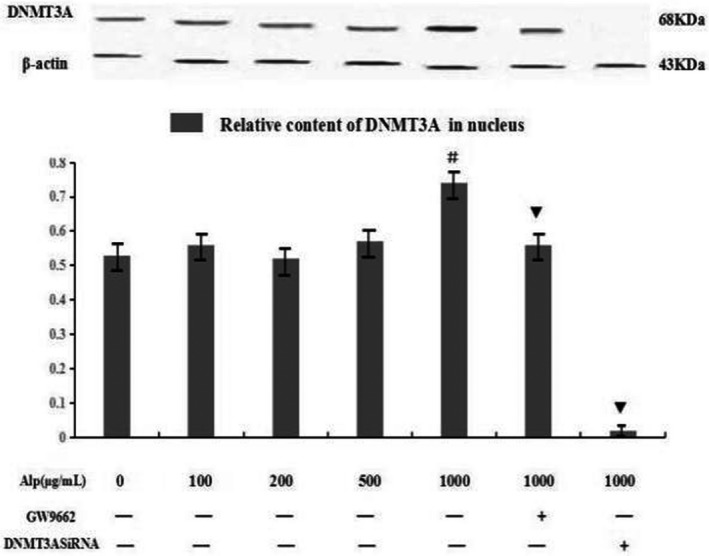
Effect of alpinetin on protein expression of DNMT3A. Expressions of PPAR and DNMT3A in RAW246.7 cells were determined by Western blot assay after cells were treated with alpinetin (0, 50, 100, 200, 500, 1,000 g/ml). Data were expressed as means ± *SD*. ^#^ *p* < .05 versus control group, ^▼^ *p* < .05 versus 1 mg/ml Alp group

Furthermore, protein expression of DNMT3A in RAW246.7 cells was estimated by Western blot analysis. Data in Figure 5 showed that alpinetin at a high concentration (1,000 μg/ml) increased the protein expression level of DNMT3A (*p* < .05), while alpinetin at the initial concentration below 500 μg/ml showed no effects on the content of DNMT3A (*p* > .05). This promoting effect could be completely reversed by interference of DNMT3A and partially blocked by GW9662.

**Figure 6:**
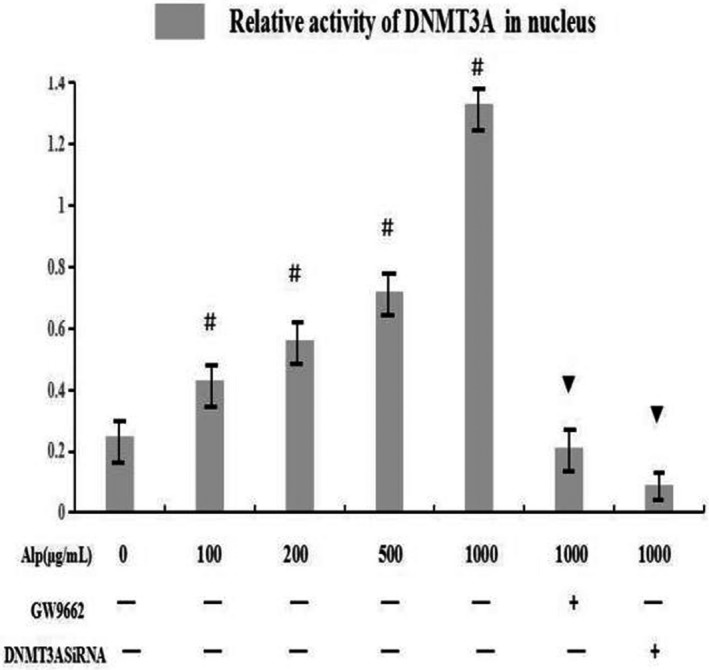
Effect of alpinetin on DNMT3A activity. DNMT3A activities were confirmed by 3‐methyltyrosine‐to‐tyrosine conversion experiment in RAW246.7 cells. Dates were expressed as means ± *SD*. ^#^ *p* < .05 versus control group, ^▼^ *p* < .05 versus 1mg/ml Alp group

DNMT3A activity was also evaluated to support above results based on the peak height ratio of 3‐methyl tyrosine to tyrosine in mass spectrometry. Results in Figure 6 showed that alpinetin at different concentrations (0, 50, 100, 200, 500, 1,000 g/mL) promoted the activity of DNMT3A in nucleus and this effect showed a dose‐dependent manner (*p* < .05). This increase could be reversed by the use of GW9662 or interference of DNMT3A, suggesting that DNMT3A was the methyltransferase activated following the alpinetin‐induced activation of PPAR.

**Figure 7:**
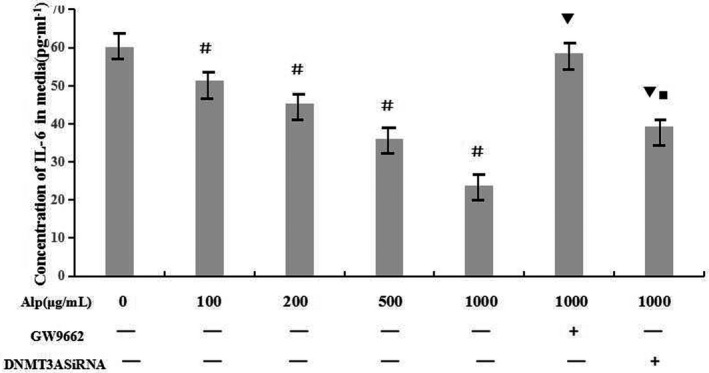
Effect of alpinetin on IL‐6 production. IL‐6 level in the culture medium of RAW246.7 cells was determined by ELISA. Data were expressed as means ± *SD*. ^#^ *p* < .05 versus control group, ^▼^ *p* < .05 versus 1 mg/ml Alp group, ^■^ *p* < .05 versus 1 mg/ml Alp + GW9662 group

The levels of IL‐6 secreted by RAW246.7 cells in all groups were evaluated by ELISA. Data in Figure 7 demonstrated that alpinetin down‐regulated IL‐6 expression in a dose‐dependent manner (*p* < .05) and this decline could be completely blocked if GW9662 was added in advance. Interference of DNMT3A could also reverse the effect caused by alpinetin, but the extent was lowered compared with GW9662.

### 3.4 The methylation ratio of CpG dinucleotide in the IL‐6 promoter region was negatively correlated with IL‐6 level

**Figure 8:**
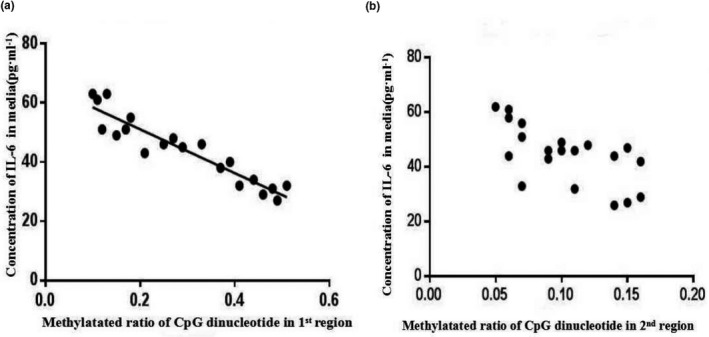
Relationship between the methylation ratio of CpG dinucleotide in IL‐6 promoter region and the IL‐6 level. The correlation between the methylation ratio of CpG dinucleotide at the first (a) and second (b) CpG islands and IL‐6 level (pg/ml) were analyzed

Linear correlation analysis was applied to assess the association between the methylation ratio of CpG dinucleotide located at the two CpG islands and IL‐6 level in the culture medium of RAW246.7 cells. As demonstrated in Figure 8, a significantly negative association was found between the methylation ratio at the first CpG island and IL‐6 level (regression equation: *y*=−74.02*x* + 65.89, *r* = −0.879, *p* < .01). However, no obvious relevance was confirmed between the IL‐6 level and the methylation ratio at the second CpG island (*p* > .05).

## 4 DISCUSSION

At present, inflammatory diseases are the major threat to human health and affect millions of people worldwide (Abdelhalim et al., 2018). Although hormones exhibit a considerable inhibitory effect on non‐specific inflammations, their side effects should not be neglected. Previous studies provided the evidences that alpinetin has protective effects on acute pulmonary injury, ulcerative colitis and atherosclerosis (AS) in animal models (Jiang, Sang, Fu, Liang, & Li, 2015; Liang et al., 2016; Zhou et al., 2008). Further understanding on the anti‐inflammatory effects and mechanism of alpinetin in RAW246.7 cells can help us find a better treatment for inflammatory diseases.

Researchers have already conducted a full research into the anti‐inflammatory mechanism of alpinetin and found that the nuclear factor kappa B (NF‐кBs) and extracellular regulates protein kinases (ERKs) signaling pathways are inhibited after alpinetin activates PPAR, thus leading to a reduced synthesis of inflammatory factors (Liu et al., 2017; Ma et al., 2017). However, this is far from being a sufficient explanation for the inhibited expression of inflammatory factors after the activation of PPAR. Along with the emergence of the epigenetic detection techniques, some research teams have found that acetylation is involved in the expression of inflammatory mediators and progression of the inflammatory diseases (Kim et al., 2015; Zhang et al., 2010). In this study, we detected intranuclear deacetylase activity in RAW246.7 cells following alpinetin treatment and proved that the increased activity of DNMT3A is related to the deacetylation of CpG binding to the promoter region of the inflammatory factors. Such deacetylation further results in the disorder of the transcriptional factor binding to the promoter, which finally influences the expression of the inflammatory factors. However, interference experiment indicated that PPAR antagonist GW9662 can completely reverse the alpinetin‐induced interference to the synthesis of the inflammatory factors. In contrast, interference of DNMT3A only partially reverses the effect of alpinetin on the synthesis of the inflammatory factors, implying that alpinetin inhibits the expression of the inflammatory factors via the activation of PPAR.

DNA methylation is the most widely studied epigenetic mechanism. It is generally accepted that abnormal methylation of cytosine or histone is involved in the occurrence and development of inflammatory diseases and tumors (Shi et al., 2018). For example, the methylation level of IL‐6 gene can affect its protein expression, which further induces inflammatory responses in both cord blood monocytes and SK‐N‐BE neuroblastoma cells (Dinicola, Proietti, Cucina, Bizzarri, & Fuso, 2017; Sureshchandra et al., 2017). In animals with AS, the average methylation level at several CpG sites in the core regulatory region of monocyte TLR4 promoter is decreased. Moreover, the H3K27Me3 level in the blood vessel plaques correlates to AS, and the expressions of methyltransferases MLL2, G9a and DNA methyltransferase 1 (DNMT1) are increased in unstable plaques (Greiβel et al., 2016; Wierda et al., 2015). Transfecting the interference fragment that contains enhancer of zeste homologue 2 (EZH2) into the macrophages will cause a reduction in the H3K27 methylation level, which further inhibits the methylation of the integral membrane protein ATP‐binding cassette transporter A1 (ABCA1) promoter and promotes the expression of ABCA1. As a result, the lipids will be transported out of the cells, which helps stabilize the AS plaques (Liang et al., 2013). In addition, methyltransferase inhibitor 5‐Aza‐Cdr inhibits the synthesis of inflammatory factors in endothelial cells induced by shock (Di Taranto et al., 2012), which also delays the formation of AS plaques in ApoE‐knockout mice (Cao et al., 2014). Taken together, methylation is involved in inflammatory factor synthesis, occurrence and development of inflammatory diseases and drug intervention of the inflammatory response. Methylation may be also the key mechanism for PPAR agonist inhibiting the expression of the inflammatory factors. To verify above hypotheses, we performed co‐immunoprecipitation assay in preliminary experiment, which indicated that alpinetin promotes the binding of PPAR to DNMT3A. DNMT3A is composed of the N‐terminal domain and C‐terminal with methyltransferase activity and is the most important methyltransferase for CpG islands in mammals (Tajima, Suetake, Takeshita, Nakagawa, & Kimura, 2016). Also, this protein catalyzes the conversion of cytosine into 5‐methylcytosine to influence gene expression (Cole et al., 2017; Yang et al., 2017). IL‐6 is found to be the most important B‐cell activating factor back in the 20th century, which mediates the cross‐linking between several immunocytes and function execution and acts as the key factor in triggering the inflammatory cascade (Ohtsu et al., 2017). We verified that alpinetin first activates PPAR and then promotes cytosine methylation of the IL‐6 promoter region by activating DNMT3A, thereby regulating the expression of IL‐6 in RAW246.7 cells.

## 5 CONCLUSIONS AND LIMITATIONS

In summary, alpinetin‐induced PPAR activation further increases DNMT3A activity or promotes its synthesis in RAW246.7 cells. As a result, cytosine methylation of the CpG islands in the IL‐6 promoter is promoted and the expression of IL‐6 is inhibited. Our study provides a new anti‐inflammatory mechanism of alpinetin from the methylation perspective, and indicates that reversing DNA methylation may be the new orientation for treating inflammatory diseases with alpinetin and other Dong medicines. However, there are several limitations. First, the RAW246.7 cells line represents a major limit, results in this study needs to be repeated in other cell lines. Second, we only investigate the effects of alpinetin on inflammatory disease in cultured cells, and a possible clinical treatment for alpinetin in animals remains to be ascertained.

## CONFLICT OF INTEREST

All authors declare that they have no conflict of interest.

## ETHICAL APPROVAL

This article does not contain any studies with human participants performed by any of the authors.
